# Precancerous Lesions and Liver Atrophy as Risk Factors for Hepatolithiasis-Related Death after Liver Resection for Hepatolithiasis

**DOI:** 10.31557/APJCP.2020.21.12.3647

**Published:** 2020-12

**Authors:** Toru Miyazaki, Hiroji Shinkawa, Shigekazu Takemura, Shogo Tanaka, Ryosuke Amano, Kenjiro Kimura, Go Ohira, Kohei Nishio, Masahiko Kinoshita, Jun Tauchi, Atsushi Ishihara, Shimpei Eguchi, Daisuke Shirai, Takatsugu Yamamoto, Kenichi Wakasa, Norifumi Kawada, Shoji Kubo

**Affiliations:** 1 *Department of Hepato-Biliary-Pancreatic Surgery Osaka City University Graduate School of Medicine 1-4-3 Asahimachi, Abenoku, Osaka 545-8585, Japan. *; 2 *Department of Surgery, Minamitama Hospital, 3-10-1 Sandamachi, Hachioji, Tokyo 193-0832, Japan. *; 3 *Department of Pathology, Ishikiriseiki Hospital, 18-28 Yayoicho, Higashiosaka, Osaka, 579-8026, Japan. *; 4 *Department of Hepatology, Osaka City University Graduate School of Medicine 1-4-3 Asahimachi, Abenoku, Osaka, Japan. *

**Keywords:** Biliary cirrhosis, biliary intraepithelial neoplasia, cholangiocarcinoma, hepatolithiasis, liver atrophy

## Abstract

**Background:**

Cholangiocarcinoma and secondary biliary cirrhosis can develop after liver resection for hepatolithiasis and are causes of hepatolithiasis-related death. We determined potential risk factors for hepatolithiasis-related death and subsequent cholangiocarcinoma, including precancerous lesions such as biliary intraepithelial neoplasia (BilIN) and intraductal papillary neoplasm of the bile duct, in patients undergoing liver resection for hepatolithiasis.

**Methods:**

The study cohort included 62 patients who underwent liver resection for hepatolithiasis without concomitant cholangiocarcinoma and had surgical specimens available for pathological examination. Univariate and multivariate analyses were conducted to examine risk factors associated with subsequent cholangiocarcinoma after hepatolithiasis and hepatolithiasis-related death. In 28 patients with BilIN lesions, the specimens were immunohistochemically stained for γ-H2AX and S100P.

**Results:**

In the study cohort, the causes of death were subsequent cholangiocarcinoma, biliary cirrhosis, and other diseases in 5, 3, and 7 patients, respectively. Liver atrophy, precancerous lesions, postoperative repeated cholangitis, and jaundice for ≥1 week during the follow-up period were risk factors for hepatolithiasis-related death. Multivariate analysis showed that liver atrophy and precancerous lesions were independent risk factors for hepatolithiasis-related death. Liver atrophy or precancerous lesions were also risk factors for subsequent cholangiocarcinoma by univariate analysis. The positive expression of γ-H2AX and S100P was observed in 18 and 14 of the 28 BilIN lesions, respectively.

**Conclusions:**

Liver atrophy and precancerous lesions with malignant transformation were risk factors not only for subsequent cholangiocarcinoma but also hepatolithiasis-related death after liver resection for hepatolithiasis, indicating that long-term follow-up is necessary even after liver resection in patients harboring these risk factors.

## Introduction

Albeit a benign disease, hepatolithiasis can lead to cholangiocarcinoma and secondary biliary cirrhosis due to repeated cholangitis and stones (Kubo et al., 1995; Kim et al., 2015a; Suzuki et al., 2018). Therefore, complete removal of hepatolithiasis and elimination of bile stasis due to bile duct strictures are necessary for the prevention of repeated cholangitis and stone recurrence. As the most effective treatment of hepatolithiasis, liver resection can remove both the intrahepatic stones and the bile duct with strictures responsible for repeated cholangitis and stone formation (Uenishi et al., 2009). However, secondary biliary cirrhosis and cholangiocarcinoma, which can develop even after liver resection in some patients, have been reported as unfavorable prognostic factors (Suzuki et al., 2012; Kim et al., 2015b).

Biliary intraepithelial neoplasia (BilIN) and intraductal neoplasm of the bile duct (IPNB), which are considered as precancerous or early cancerous lesions (WHO, 2019), are observed in hepatolithiasis, primary sclerosing cholangitis, and occupational cholangiocarcinoma caused by chlorinated organic solvents (Chen et al., 2001; Itatsu et al., 2007; Kinoshita et al., 2016). Chronic inflammation causes malignant transformation of these precancerous or early cancerous lesions, eventually leading to invasive cholangiocarcinoma (Nakanuma et al., 2009).

Although risk factors for hepatolithiasis-related death and subsequent cholangiocarcinoma after liver resection for hepatolithiasis were reported by a few studies (Kim et al., 2015b; Suzuki et al., 2018), precancerous lesions such as BilIN and IPNB have not been evaluated. In the present study, we investigated risk factors, including precancerous lesions, for hepatolithiasis-related death and subsequent cholangiocarcinoma after liver resection for hepatolithiasis.

## Materials and Methods

*Patients*

Surgical specimens of 72 patients who underwent liver resection for hepatolithiasis at the Department of Hepato-Biliary-Pancreatic Surgery in Osaka City University Hospital between 1978 and 2017 were available for pathological examination. Of these, nine patients with concomitant cholangiocarcinoma and one patient who died of bacterial endocarditis after surgery (in-hospital death) were excluded from the study. Therefore, the study cohort included the remaining 62 patients. The median follow-up period from surgery until death or the end of the study (July 31, 2019) was 3870 (range, 389–10958) days. This study was conducted following the guidelines of the Declaration of Helsinki and was approved by the Ethics Committee of Osaka City University Graduate School of Medicine (No. 4013).

*Outcomes and prognostic factors after liver resection*

The outcomes after liver resection for hepatolithiasis and prognostic factors were assessed by survival rates after liver resection. The causes of death were also investigated. Variables potentially associated with survival were based on the results of previous studies and our clinical experience (Liu et al., 2011; Suzuki et al., 2014; Kim et al., 2015b). The following variables were included in the present study: age (<65 vs ≥65 years), sex, symptom duration (≤10 vs >10 years), history of cholecystectomy, history of choledocho-enterostomy, serum levels of carcinoembryonic antigen and carbohydrate antigen 19-9 (CA 19-9), preoperative cholangitis, stone location, bile duct stenosis, bile duct dilatation, type of surgical procedure, biliary reconstruction, liver atrophy of affected segment(s) defined as more than 50% reduction in liver volume on computed tomography (CT) or magnetic resonance imaging (MRI) (Ham, 1979), precancerous lesions (BilIN and IPNB), residual stones after surgery, stone recurrence, jaundice for ≥1 week during the follow-up period, and postoperative repeated cholangitis. Patients who were administered oral or intravenous antibiotics for cholangitis twice or more per year and those with stone recurrence caused by cholangitis were classified as those with repeated cholangitis (Aota et al., 2019).

*Pathological examination*

Pathological findings were evaluated according to the World Health Organization classification for digestive system tumors (WHO, 2019). Briefly, BilIN lesions were pathologically classified as low-grade BilIN and high-grade BilIN according to the cellular and structural features. Low-grade BilIN was characterized by mild cytoarchitectural atypia including a predominantly flat growth pattern, pseudostratification of nuclei, and a high nucleus/cytoplasm ratio. High-grade BilIN was characterized by more complex patterns, such as micropapillae and tall papillae. IPNB was defined as a grossly visible premalignant lesion with intraductal papillary or villous growth of biliary epithelium.

The surgical specimens were available for immunohistochemical examination in 28 of the 29 patients with BilIN or IPNB lesions. The specimens were immunohistochemically stained for γ-H2AX and S100P. Immunohistochemical staining was performed using primary antibodies against S100P (rabbit monoclonal antibody, ab133554; Abcam, Cambridge, England) and γ-H2AX (rabbit monoclonal antibody, ab81299; Abcam), as reported previously (Kinoshita et al., 2016). The tissue sections were deparaffinized with xylene and rehydrated through ethanol series and phosphate-buffered saline. Antigen retrieval was performed by microwave treatment using a citrate buffer (pH 6). Endogenous peroxidase was blocked with 0.3% H_2_O_2_ in methanol for 30 min, followed by incubation with G-Block (Genostaff, Tokyo, Japan) and the avidin/biotin blocking kit (Vector, Burlingame, USA). The sections were incubated with the anti-S100P or γ-H2AX rabbit monoclonal antibody overnight at 4^o^C, followed by a biotin-conjugated anti-rabbit IgG (Dako, Santa Clara, USA) for 30 min at room temperature and the addition of peroxidase-conjugated streptavidin (Nichirei, Tokyo, Japan) for 5 min at room temperature. Peroxidase activity was visualized by diaminobenzidine. The sections were counterstained with Mayer’s hematoxylin (MUTO, Tokyo, Japan), dehydrated, and mounted with Malinol mounting medium (MUTO). Multiple fields in sections were observed under ×200 magnification, and areas with strongest nuclear staining for γ-H2AX and S100P were selected to determine the proportions of cells that were positive and negative for γ-H2AX and S100P.

*Statistical analysis*

Continuous variables were compared using Student’s *t *test, and categorical variables were compared using Fisher’s exact test. Cumulative survival rates were calculated by the Kaplan-Meier method and compared by the log-rank test. The Cox proportional hazards model with stepwise variable selection was used for multivariate analysis. Factors with a *P* value of <0.1 in the univariate analysis were entered into the multivariate analysis. A *P* value of <0.05 was considered to indicate statistical significance. All analyses were performed using EZR.

## Results

*Characteristics of patients who died of hepatolithiasis-related causes*

In the present study, 5 of the 62 patients developed subsequent cholangiocarcinoma and 15 of the 62 patients died during the follow-up period. The causes of death were subsequent cholangiocarcinoma after liver resection, secondary biliary cirrhosis, pneumonia, and other malignancies in 5, 3, 2, and 5 patients, respectively. Therefore, 8 of 62 patients died of hepatolithiasis-related causes including cholangiocarcinoma and secondary biliary cirrhosis.

**Figure 1:**
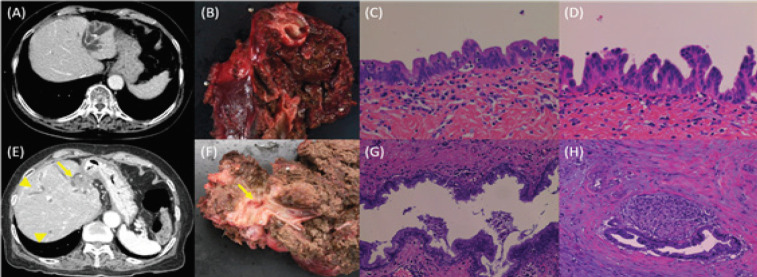
Representative Computed Tomography Images, Surgical Specimens, and Pathological Findings of One Patient (Patient No. 5) with Subsequent Cholangiocarcinoma after Liver Resection for Hepatolithiasis. (A) Computed tomography image before the first surgery shows intrahepatic stones, bile duct dilation, and liver atrophy. (B) Prominent bile duct dilation and liver atrophy are observed in macroscopic examination of the specimen resected in the first operation. (C) Low-grade biliary intraepithelial neoplasia (BilIN) is observed in the surgical specimen of the first operation. Hematoxylin and eosin (HE) staining. Magnification, ×400. (D) High-grade BilIN is observed in a surgical specimen of the first operation. HE staining. Magnification, ×400. (E) Computed tomography image before the second surgery shows cholangiocarcinoma (arrow) adjacent to the stump of the resected bile duct (the branching point of the bile duct of the medial segment and left hepatic duct). The dilatation of the intrahepatic bile ducts in the right lobe (arrow head) were also observed. (F) Bile duct dilation and cholangiocarcinoma (arrow) are observed in macroscopic examination of the specimen resected in the second operation. (G) High-grade BilIN is observed in the surgical specimen resected in the second operation. HE staining. Magnification, ×200. (H) Cholangiocarcinoma (well-differentiated adenocarcinoma) in the surgical specimen resected in the second operation

The characteristics of the five patients with subsequent cholangiocarcinoma are summarized in Table 1. Cholangiocarcinoma was diagnosed between 2 years 3 months and 18 years after the liver resection; the median time from surgery to cholangiocarcinoma diagnosis was ten years. The site of cholangiocarcinoma was adjacent to the stump of the resected bile duct in all patients. The representative CT images, surgical specimens, and pathological findings of Patient No. 5 are shown in Figure 1. The patient underwent left lateral sectionectomy for hepatolithiasis in the lateral segment. The high- and low-grade BilIN lesions were detected in the resected liver. A space-occupying lesion observed in CT images indicated the subsequent development of cholangiocarcinoma in the left hepatic duct at 3 years 9 months after the first operation. Although medial segmentectomy was performed, curative resection could not be performed because the intrahepatic bile ducts were involved. The pathological examination of the surgical specimen revealed cholangiocarcinoma with periductal invasion and BilIN lesions. She died of the remnant cholangiocarcinoma at 3 months after the second operation.

Three patients died of secondary biliary cirrhosis. One patient with intrahepatic and extrahepatic stones in bilateral lobes with a history of cholecystectomy underwent left lobectomy because the left lobe was severely atrophied. However, the patient had repeated cholangitis with jaundice due to residual stricture of the bile duct and residual stones in the right lobe. The remaining two patients had repeated cholangitis with jaundice for ≥1 week and stone reformation due to the residual stricture of the bile duct in the remnant liver.

All eight patients with symptoms due to stone or bile duct stricture after surgery, except two treated in other hospitals and one for which no records were available, underwent treatment for stone removal or improvement of bile duct stricture by percutaneous transhepatic cholangioscopy, endoscopic retrograde cholangiography, or surgery. However, the above mentioned three patients did not respond well to treatment due to diffuse stones and bile duct stenosis/dilatation in the remaining liver, leading to secondary biliary cirrhosis. In the other five patients, our treatment improved their symptoms. Moreover, no stone recurrence was found in patients who underwent biliary reconstruction in our surgery.

*Risk factors for hepatolithiasis-related death*

**Figure 2:**
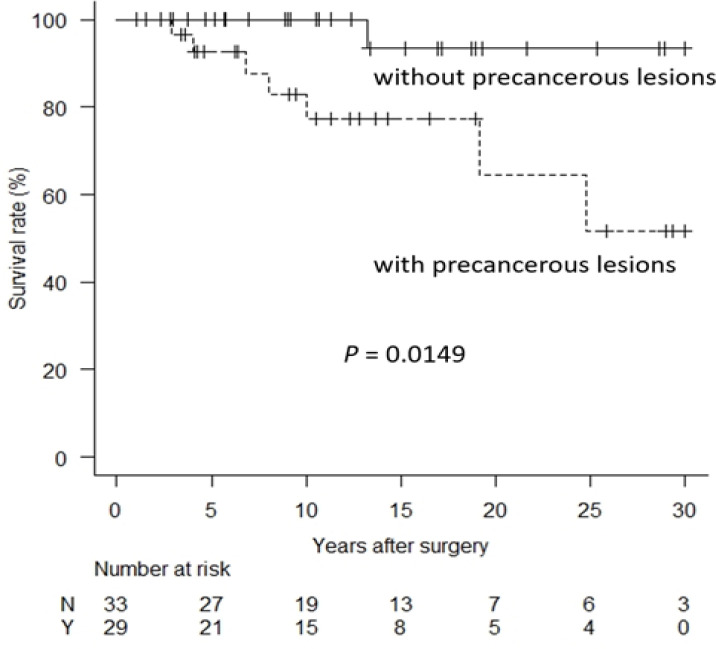
Hepatolithiasis-Related Death-Free Survival Rates in Patients with and without Precancerous Lesions

Risk factors for hepatolithiasis-related death were evaluated by univariate and multivariate analyses, which revealed that liver atrophy, precancerous lesions, postoperative repeated cholangitis, and jaundice for ≥1 week during the follow-up period were risk factors for hepatolithiasis-related death (Table 2). The hepatolithiasis-related death-free survival rate was significantly lower in the patients with precancerous lesions compared to those without the lesions (*P* = 0.0149, Figure 2). Jaundice for ≥1 week during the follow-up period was included as a potential risk factor in the multivariate analysis because the patients with jaundice for ≥1 week during the follow-up period overlapped with those who developed postoperative repeated cholangitis. The multivariate analysis showed as follows; liver atrophy (risk ratio = 10.4, 95% confidence interval = 1.69–64.2, *P* value = 0.011), precancerous lesions (risk ratio = 12.7, 95% confidence interval = 1.36–118, *P* value = 0.026), and postoperative jaundice (risk ratio = 0.294, 95% confidence interval = 0.0631–1.37, *P* value = 0.12) (Supplementary Table 1). These results indicated that liver atrophy and precancerous lesions were independent risk factors for hepatolithiasis-related death.

*Risk factors for subsequent cholangiocarcinoma*

The characteristics of patients with and without subsequent cholangiocarcinoma are summarized in Table 3. Briefly, the proportions of patients with liver atrophy or precancerous lesions were significantly higher among patients with subsequent cholangiocarcinoma than in those without subsequent cholangiocarcinoma.

*Precancerous lesions in hepatolithiasis*

**Figure 3:**
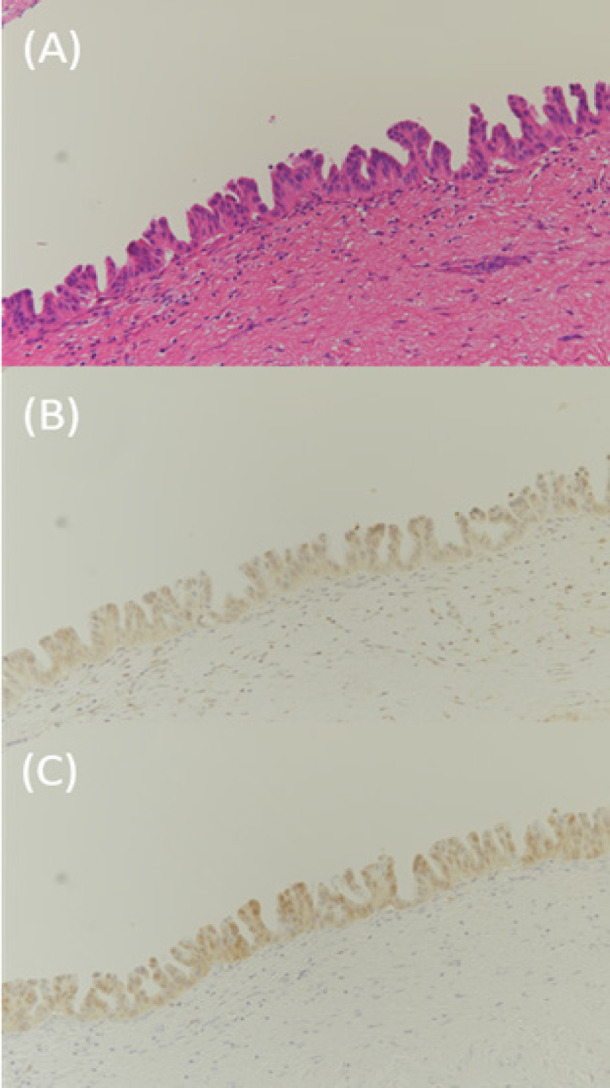
Immunohistochemical Staining of Biliary Intraepithelial Neoplasia Specimens by γ-H2AX and S100P. Magnification, ×200. (A) Biliary intraepithelial neoplasia. HE staining. (B) Immunohistochemical staining for γ-H2AX. (C) Immunohistochemical staining for S100P

By pathological examination, BilIN and IPNB were found in 28 and 1 patient, respectively. The results of the immunohistochemical staining to assess the positive expression of γ-H2AX and S100P in the BilIN lesions of 28 patients are shown in Fig. 3. The positive expression of γ-H2AX was observed in 18 patients, and the positive expression of S100P was observed in 14 patients, indicating the presence of DNA injury and malignant transformation in many of the BilIN lesions.

All five patients who developed subsequent cholangiocarcinoma had BilIN or IPNB lesions, and the positive expressions of γ-H2AX and S100P were detected in the BilIN lesions of four patients. The surgical specimen of IPNB lesions was not available. In contrast, patients without γ-H2AX or S100P expressions did not develop postoperative carcinogenesis. Two of three patients who died of secondary biliary cirrhosis had BilIN lesions, and the positive expressions of γ-H2AX and S100P were detected in the BilIN lesions of one of the two patients.

**Table 1:** Characteristics of Five Patients with Subsequent Cholangiocarcinoma

	First operation								Cholangiocarcinoma				
Patient No.	Age	Sex	Operative procedure	Liver atrophy	Residual stones	BilIN or IPNB	Expression of γ-H2AX	Expression of S100P	Duration between first surgery and cholangiocarcinoma diagnosis	Site	Stage	Treatments	Duration between diagnosis of cholangiocarcinoma and mortality
1	48	M	Right posterior sectionectomy and Segmentectomy 3	Positive	Negative	low-grade BilIN	Positive	Positive	18 years	Right hepatic duct - extrahepatic bile duct	IIIA	Radiation	1 year
2	45	M	Right hepatectomy and Segmentectomy 3	Positive	Positive	low-grade BilIN	Positive	Positive	2 years 3 months	Left hepatic duct - medial segment, bone metastasis	IVB	Conservative	7 months
3	57	F	Left hepatectomy with segmentectomy 1	Positive	Negative	low-grade IPNB	NA‡	NA‡	17 years	Extrahepatic bile duct	II	Resection of extrahepatic bile duct and hepaticojejunostomy, adjuvant chemotherapy	7 years†
4	58	M	Left hepatectomy with segmentectomy 1	Positive	Negative	low-grade BilIN	Positive	Positive	10 years	Extrahepatic bile duct	IVA	Conservative	4 months
5	82	F	Left lateral sectionectomy	Positive	Negative	high-grade BilIN	Positive	Positive	3 years 9 months	Left hepatic duct - medial segment	IVA	Segmentectomy 4	3 months

BilIN, biliary intraepithelial neoplasia; IPNB, intraductal papillary neoplasm of the bile duct; M, male; F, Female; †, The chemotherapy was performed for the recurrence of cholangiocarcinoma that was detected at 5 years and 7 months after the operation for subsequent cholangiocarcinoma; ‡, The surgical specimens were not available.

**Table 2:** Univariate Analysis of Risk Factors for Hepatolithiasis-Related Death Using the Log-Rank Test

Variables	Number	Survival rate (%)			P value
		3 years	7 years	10 years	
Age, years					
<65	43	98	94	91	0.627
≥65	19	100	94	84	
Sex					
Male	22	95	88	81	0.164
Female	40	100	97	97	
Period from onset to surgery (years)					
<10	36	100	92	86	0.576
≥10	26	96	96	92	
History of cholecystectomy					
Present	21	100	100	100	0.0974
Absent	41	97	95	84	
History of choledocho-enterostomy					
Present	3	100	100	100	0.694
Absent	59	98	96	89	
CA19-9 (U/mL)					
<37	34	100	97	86	0.97
≥37	7	86	86	86	
Preoperative cholangitis					
Present	3	100	100	100	0.714
Absent	42	97	95	87	
Bile duct stenosis					
Present	51	98	96	88	0.289
Absent	11	100	100	100	
Biliary reconstruction					
Present	10	100	100	83	0.454
Absent	52	98	93	90	
Liver atrophy of affected segment (s)					
Present	16	94	87	70	0.000599
Absent	46	100	97	97	
Precancerous lesions †					
Present	29	96	88	77	0.0149
Absent	33	100	100	100	
Residual stones after surgery					
Present	8	88	88	88	0.49
Absent	54	98	95	89	
Stone recurrence					
Present	5	100	75	50	0.162
Absent	56	98	96	93	
Postoperative repeated cholangitis					
Present	13	100	89	78	0.00942
Absent	49	98	95	92	
Postoperative jaundice					
Present	14	100	80	80	0.00831
Absent	48	98	95	91	

†, Precancerous lesions include biliary intraepithelial neoplasia and intraductal papillary neoplasm of the bile duct; CA19-9, carbohydrate antigen 19-9

**Table 3:** Characteristics of Patients with and without Subsequent Cholangiocarcinoma

	Hepatolithiasis without subsequent cholangiocarcinoma (n = 57)	Hepatolithiasis with subsequent cholangiocarcinoma (n = 5)	P value
Age > 65 years	18/57	1/5	0.999
Sex (M/F)	19:38	3:02	0.337
Period from onset to surgery (>10 years)	23/57	3	0.641
History of cholecystectomy	21/57	0/5	0.157
History of choledocho-enterostomy	3/57	0/5	>0.999
Tumor marker levels immediately before surgery			
CEA > 5.0 ng/mL	0/51 †	0/5	
CA19-9 > 37 U/mL	6/36 †	1/5	>0.999
Preoperative cholangitis	37/40 †	5/5	>0.999
Location of stones			0.667
Right	10	0	
Left	34	3	
Both	13	2	
Bile duct stenosis	46/57	5/5	0.575
Bile duct dilatation	31/57	3/5	>0.999
Operative procedure			0.651
Right hepatectomy ‡	5	1	
Left hepatectomy ‡	18	2	
Left lateral sectionectomy	23	1	
Other type resections	11	1	
Biliary reconstruction	9/57	1/5	>0.999
Liver atrophy of affected segment(s)	12/57	4/5	0.0136
Precancerous lesions ‡†	24/57	5/5	0.0184
Residual stones after surgery	7/57	1/5	0.511
Stone recurrence	5/57	0/5	>0.999
Postoperative repeated cholangitis	11/57	2/5	0.28
Postoperative jaundice	11/57	3/5	0.0712

†, Patients for which details of variable were not be available were excluded; ‡, Right or left hepatectomy with or without partial resection of the other part of the liver; ‡†, Precancerous lesions included biliary intraepithelial neoplasia and intraductal papillary neoplasm of the bile duct; CEA, carcinoembryonic antigen; CA19-9, carbohydrate antigen 19-9

## Discussion

The present study revealed that liver atrophy, precancerous lesions, repeated cholangitis, and jaundice for ≥1 week during the follow-up period were risk factors by univariate analysis and that liver atrophy and precancerous lesions were independent risk factors for hepatolithiasis-related death (cholangiocarcinoma and secondary biliary cirrhosis) by multivariate analysis. Furthermore, liver atrophy and precancerous lesions were also risk factors for subsequent cholangiocarcinoma by univariate analysis.

The development of cholangiocarcinoma in patients with hepatolithiasis is a critical issue. Although the observation period has varied among the studies published to date, the reported incidence rates of subsequent cholangiocarcinoma after any treatments are 4.8% (11/227 patients) (Cheon et al., 2009), 6.8% (16/236 patients) (Kim et al., 2015b), and 6.9% (27/392 patients) (Suzuki et al., 2018). Furthermore, the reported incidence rates of subsequent cholangiocarcinoma after liver resection are 4% (3/77 patients during a median 8-year follow-up period) (Cheon et al., 2009), and 6.3% (6/95 patients during a median 41-month follow-up period) (Kim et al., 2015b). In the present study, subsequent cholangiocarcinoma developed in 5 of the 62 patients (8.1%). The slightly higher rate of subsequent cholangiocarcinoma after treatment for hepatolithiasis might be related to the long-term observation period in the present study.

Several studies have previously reported old age, bile duct stenosis, bilioenteric anastomosis, stone recurrence, stones in both hepatic lobes, and no liver resection as risk factors for subsequent cholangiocarcinoma after treatment (Li et al., 2012; Suzuki et al., 2012; Kim et al., 2015b; Suzuki et al., 2018). In the present study, liver atrophy and precancerous lesions, which have not been evaluated in previous studies, were significant risk factors for subsequent cholangiocarcinoma after treatment for hepatolithiasis. Repeated cholangitis and chronic inﬂammation of the biliary epithelium have been reported to lead to precancerous or early cancerous lesions, with subsequent cholangiocarcinoma in a multistep fashion (Nakanuma et al., 2009). Itatsu et al., (2007) showed that BilIN was observed in 36 of 55 patients with hepatolithiasis without cholangiocarcinoma and in 9 of 19 patients with hepatolithiasis and concomitant cholangiocarcinoma; the authors also reported that IPNB was observed in 9 of the 55 patients and 10 of the 19 patients. In the present study including 62 patients, BilIN and IPNB were found in 28 and 1 patient, respectively. Importantly, all five patients with subsequent cholangiocarcinoma exhibited these precancerous lesions.

The positive expression of γ-H2AX, the phosphorylated form of a histone 2A variant which indicates the presence of DNA double-stranded breaks, i.e., DNA injury, is observed in various cancers (Bartkova et al., 2005). The positive expression of γ-H2AX was previously reported in precancerous lesions such as BilIN as well as cholangiocarcinoma in patients with hepatolithiasis and occupational cholangiocarcinoma (Kinoshita et al., 2016). S100P, a member of the S100 family, has been shown to mediate malignant transformation and progression (Jiang et al., 2012). Previous studies have shown that S100P is expressed in cholangiocarcinoma and BilIN lesions in patients with hepatolithiasis (Sato et al., 2013). In this study, immunohistochemical evaluation revealed the presence of γ-H2AX and S100P in 18 and 14 of the 28 BilIN lesions, respectively. Furthermore, γ-H2AX and S100P expressions were detected in all BilIN lesions in patients with subsequent cholangiocarcinoma. On the other hand, patients without γ-H2AX or S100P expressions did not develop postoperative carcinogenesis. These results suggested that chronic inflammation might have led to DNA injury and malignant transformation of the bile duct epithelium. In the present study, the relationship between precancerous lesions and liver atrophy was unclear although both were risk factors for subsequent cholangiocarcinoma. For the development of cholangiocarcinoma, DNA injury and the abovementioned genetic alternations might be necessary in addition to liver atrophy.

In the present study, cholangiocarcinoma was detected between 2 years 3 months and 18 years after the liver resection in five patients. The site of the cholangiocarcinoma was adjacent to the margin of the resected bile duct in all patients. Kusano et al., (2001) reported that subsequent cholangiocarcinoma after liver resection for hepatolithiasis occurred within the same hepatic lobe where treatment was performed in most of the cases. Nakanuma et al., (2003) reported that chronic inflammatory conditions played a role in the development of cholangiocarcinoma arising from the bile duct exhibiting chronic inflammatory changes. The presence of precancerous lesions suggest that precancerous or early cancerous changes might develop in the adjacent bile duct. It could be speculated that the chronic inflammatory conditions in the resected bile ducts had spread to the adjacent nonatrophic bile ducts and that these inflammations had induced precancerous lesions or carcinogenesis in the bile ducts in the adjacent segments. In bile ducts not adjacent to the stump, the risk of carcinogenesis might be low because inflammation had not spread to the segments away from the surgical stump.

Our study indicates that close follow-up for an extended time period to assess for the development of cholangiocarcinoma is necessary to detect cholangiocarcinoma at an early stage. In the cases of other cholangiocarcinoma risk factors such as occupational cholangiocarcinoma and primary sclerosing cholangitis, a combination of the measurement of serum concentrations of CA 19-9 and imaging examinations (ultrasonography or MRI) at 6- or 12-month intervals is recommended (Razumilava et al., 2011; Kubo et al., 2016). We suggest that follow-up observations after operation for hepatolithiasis with risk factors should be performed using the same methods as followed for these diseases. Because postoperative liver evaluation is difficult using ultrasonography, it would be better to perform imaging examinations via CT or MRI. In our study, patients after operation for hepatolithiasis were examined every 6 months for several years and every 12 months thereafter. Subsequent cholangiocarcinoma can develop more than 10 years after operation; hence, we recommend continuing the follow-up every 6 months. Although the subsequent cholangiocarcinoma developed adjacent to the stump of the resected bile duct in the present study, the necessity of extended surgical treatment for patients with hepatolithiasis and associated precancerous lesions but not for those with invasive carcinoma remains unknown.

Liver atrophy, which was reported as a prognostic factor of cholangiocarcinoma associated with hepatolithiasis, was confirmed as an independent prognostic factor in the present study (Kubo et al., 1995; Suzuki et al., 2012). Liver atrophy is related to repeated cholangitis because chronic inflammation causes portal stenosis or obstruction. Resection of the atrophied liver tissue is recommended because liver atrophy is also a risk factor for cholangiocarcinoma.

In the present study, 3 of the 62 patients (4.8%) died of secondary biliary cirrhosis due to repeated cholangitis after liver resection for hepatolithiasis. Chronic cholestasis, repeated cholangitis, and bile duct stricture lead to biliary cirrhosis (Suzuki et al., 2018). In fact, all three patients in the present study had repeated cholangitis due to residual stones and residual bile duct stricture.

The limitation of the present study was the small number of subjects and the single-hospital setting. For definitive conclusions, multicenter studies with larger number of subjects are warranted.

In conclusion, liver atrophy and precancerous lesions with malignant transformation were risk factors not only for subsequent cholangiocarcinoma but also for hepatolithiasis-related death after liver resection for hepatolithiasis. Therefore, long-term follow-up is necessary even after liver resection in patients with hepatolithiasis harboring these risk factors.
